# Chaperone Mediated Autophagy Degrades TDP-43 Protein and Is Affected by TDP-43 Aggregation

**DOI:** 10.3389/fnmol.2020.00019

**Published:** 2020-02-18

**Authors:** Fernando Ormeño, Juan Hormazabal, José Moreno, Felipe Riquelme, Javiera Rios, Alfredo Criollo, Amelina Albornoz, Iván E. Alfaro, Mauricio Budini

**Affiliations:** ^1^Dentistry Faculty, Molecular and Cellular Pathology Laboratory, Institute in Dentistry Sciences, University of Chile, Santiago, Chile; ^2^Autophagy Research Center (ARC), University of Chile, Santiago, Chile; ^3^Lysosome Biology Research Laboratory, Fundación Ciencia y Vida, Santiago, Chile; ^4^Cellular Biology Laboratory, Dentistry Faculty, Institute in Dentistry Sciences, University of Chile, Santiago, Chile; ^5^Fundación Ciencia y Vida, Santiago, Chile; ^6^Instituto de Ciencias e Innovación en Medicina, Facultad de Medicina, Clínica Alemana Universidad del Desarrollo, Santiago, Chile

**Keywords:** TARDBP, protein aggregation, cellular model, chaperone mediated autophagy, lysosomal damage, amyotrophic lateral sclerosis

## Abstract

TAR DNA binding protein 43 kDa (TDP-43) is a ribonuclear protein regulating many aspects of RNA metabolism. Amyotrophic Lateral Sclerosis (ALS) and Frontotemporal Lobar Degeneration (FTLD) are fatal neurodegenerative diseases with the presence of TDP-43 aggregates in neuronal cells. Chaperone Mediated Autophagy (CMA) is a lysosomal degradation pathway participating in the proteostasis of several cytosolic proteins including neurodegenerative associated proteins. In addition, protein oligomers or aggregates can affect the status of CMA. In this work, we studied the relationship between CMA and the physiological and pathological forms of TDP-43. First, we found that recombinant TDP-43 was specifically degraded by rat liver’s CMA+ lysosomes and that endogenous TDP-43 is localized in rat brain’s CMA+ lysosomes, indicating that TDP-43 can be a CMA substrate *in vivo*. Next, by using a previously reported TDP-43 aggregation model, we have shown that wild-type and an aggregate-prone form of TDP-43 are detected in CMA+ lysosomes isolated from cell cultures. In addition, their protein levels increased in cells displaying CMA down-regulation, indicating that these two TDP-43 forms are CMA substrates *in vitro*. Finally, we observed that the aggregate-prone form of TDP-43 is able to interact with Hsc70, to co-localize with Lamp2A, and to up-regulate the levels of these molecular components of CMA. The latter was followed by an up-regulation of the CMA activity and lysosomal damage. Altogether our data shows that: (i) TDP-43 is a CMA substrate; (ii) CMA can contribute to control the turnover of physiological and pathological forms of TDP-43; and (iii) TDP-43 aggregation can affect CMA performance. Overall, this work contributes to understanding how a dysregulation between CMA and TDP-43 would participate in neuropathological mechanisms associated with TDP-43 aggregation.

## Introduction

Ribonuclear protein TAR DNA binding protein 43 kDa (TDP-43) was first discovered as a transcription factor and a splicing regulator (Ou et al., 1995; Buratti and Baralle, 2001). Additional roles of TDP-43 in RNA metabolism processes can include pre-mRNA splicing regulation, mRNA stability, and transport, miRNA synthesis, stress granules formation, among others (Buratti and Baralle, 2009, 2012; Ratti and Buratti, 2016). In 2006, TDP-43 was found as the principal component of protein inclusions present in neuronal and non-neuronal cells of patients affected by Amyotrophy Lateral Sclerosis (ALS) and Frontotemporal Lobar Degeneration (FTLD). The role of TDP-43 aggregates is still unknown, and the hypothesis is that aggregates would affect cellular pathways by inducing gain-or loss-of-function conditions (Lee et al., 2011; Vanden Broeck et al., 2014; Budini et al., 2015). Like every protein prone to aggregate, the proteostasis of TDP-43 must be finally regulated. In this sense, many works have made important contributions to understand how the proteasome and autophagy regulate TDP-43 at both, physiologically and pathologically levels (Urushitani et al., 2010; Wang et al., 2010; Scotter et al., 2014).

Autophagy is a pathway that requires lysosomes to degrade different cellular components such as proteins or organelles (e.g., mitochondria), either to correctly maintain the basal cellular metabolism or to overcome a particular stress (e.g., nutrient deprivation, hypoxia, oxidative stress, among others; Kaushik and Cuervo, 2018). Autophagy can be divided into three different types, macroautophagy (Ma, mostly known as autophagy), endosomal microautophagy (eMi), and chaperone-mediated autophagy (CMA; Kaushik and Cuervo, 2018). Ma is able to degrade proteins (soluble and misfolded) and organelles (mitochondria, endoplasmic reticulum, lysosomes) and involves the formation of a double-membrane structure named autophagosome. Autophagosome fuses with the lysosome, forming an autolysosome, that finally destroys its content (Lamb et al., 2013). eMi is described by the selective capture of protein substrates in endosomal invaginations and multivesicular bodies (MVBs) mediated by Heat shock cognate protein 70 kDa (Hsc70), which then can fuse with the lysosomes (Sahu et al., 2011). In the case of CMA, it only degrades soluble proteins and does not require the formation of an autophagosomal structure. In this pathway, proteins to be degraded interact with the Hsc70 chaperone through a motive related to the pentapeptide KFERQ (Isenman and Dice, 1989; Dice, 1990; Wing et al., 1991; Arias and Cuervo, 2011). In contrast to eMi, Hsc70-target protein complex interacts with the lysosomal associated membrane protein isoform 2A (Lamp2A), which acts as a receptor for the translocation of the target protein to the lysosomal lumen. Thus, Hsc70 and Lamp2A are the principal players of CMA, being Lamp2A the limiting step of this process (Cuervo and Dice, 2000).

A dysregulation in autophagy has been associated with several neurodegenerative diseases (Hara et al., 2006; Komatsu et al., 2006; Guo et al., 2018). Particularly, some reports show that a dysregulation in CMA would be implicated in neurogenerative processes such as Parkinson disease (PD), Alzheimer’s disease (AD) or Huntington disease (HD). In part, this is supported by the participation of CMA in the degradation of proteins like α-Syn (alpha-synuclein; Vogiatzi et al., 2008; Xilouri et al., 2009; Mak et al., 2010), Tau (microtubule-associated protein; Wang et al., 2009, 2010; Caballero et al., 2018), LRRK2 (leucine-rich repeat kinase 2; Orenstein et al., 2013), PARK7 (Parkinson disease protein 7; Wang et al., 2016) and Htt (Huntingtin protein; Thompson et al., 2009; Koga et al., 2011a; Qi et al., 2012). In addition, abnormal forms of these proteins have been shown to affect either, positively or negatively the CMA activity, leading to a general dysfunction of this pathway (Wang et al., 2009; Xilouri et al., 2009). Although the relationship between macroautophagy with physiological and pathological forms of TDP-43 has been amply studied and reviewed (Budini et al., 2012), very little is known about the participation of CMA in controlling TDP-43 protein levels and, especially, whether TDP-43 aggregates are able to modify the status of CMA.

Here, we not only validate TDP-43 protein as a CMA substrate *in vitro*, but also suggest that CMA could participate in the degradation of TDP-43 *in vivo*. We show that the inhibition of CMA activity provokes an increase in the protein levels of wild type and an aggregate-prone form of TDP-43. Remarkably, using a previously reported cellular model (Budini et al., 2015) we observed that TDP-43 aggregation can affect CMA activity and cause lysosomal damage associated to this pathway. This work suggests that a dysregulation in CMA activity could be occurring in neuropathological mechanisms with disrupted TDP-43 proteostasis.

## Materials and Methods

### Lysosomal Isolation From Rat Tissues

Male Sprague–Dawley rats (250 *g*) were maintained with water and food *ad libitum*. All procedures were approved by the Bioethics Committee of Fundación Ciencia and Vida. Subcellular fractions enriched in intact lysosomes were prepared according to Juste and Cuervo (2019). Forty-eight hours (48 h) before the experiments, two rats were starved and then euthanized by carbon dioxide inhalation followed by cervical dislocation. Then, livers and brains were dissected and washed several times in cold 0.25 M sucrose pH 7.2 to remove any remaining blood. The two livers were processed separately, whereas the two brains were pooled for brain lysosomal isolation. Tissues were homogenized in cold room with three volumes of 0.25 M sucrose/g with a glass Teflon homogenizer. Liver or brain homogenates were filtered through double typical gauze and centrifuged at 6,800 *g* for 5 min at 4°C. Supernatants (post-nuclear fraction) were collected and centrifuged at 17,000 *g* for 10 min at 4°C. The remaining fat layer was removed from the tube walls using a clean Kim wipe and pellets were resuspended with a “cold finger” in 3.5 volumes of 0.25 M sucrose/g of tissue and centrifuged again at 17,000 *g* 10 min at 4°C. Pellets were resuspended in 0.25 M sucrose plus two volumes of 85.6% Nycodenz and loaded on the bottom of an ultra-clear ultracentrifugation tube. Then samples were centrifuged in a discontinuous Nycodenz gradient (32.8%, 26.3%, 19.8%) at 101,709 *g* for 1 h at 4°C. Four fractions were obtained from this step: 1, 2, 3 and 4 from the top to the bottom of the tube. The different fractions were collected carefully with a glass Pasteur pipette. Fractions 1 and 2 were considered as lysosomes containing high (CMA+) and low (CMA−) CMA activity, respectively. Fraction 3 was considered as a Mitochondria/lysosomal fraction, whereas faction 4 was obtained as Light Mitochondria fraction. Each sample was washed with 0.25 M sucrose by centrifugation at 37,000 *g* for 15 min at 4°C. For transport and competitive assays, liver lysosomal fractions were resuspended in 300 μl of 0.25 M sucrose pH 7.2 and used immediately (lysosomes preparations with more of 10% broken lysosomes, measured by β-hexosaminidase latency, were discarded). In the case of brain samples, all fractions were quantified, resuspended in Laemmli buffer and keep at −20°C until their analysis by Western blot.

### Lysosomal Transport and Competitive Assays

Rat liver lysosomes pre-incubated with and without protease inhibitors were mixed with MOPS-sucrose (10 mM MOPS, 0.25 M sucrose pH 7.2) in a total volume of 30 μl. Increasing amounts of recombinant TDP-43 protein (Abcam ab140718) were added. The samples were incubated at 37°C for 20 min and then centrifuged at max speed for 20 min at 4°C. Supernatants were discarded and the pellets washed with 100 μl MOPS-sucrose. For the competition assay, different amounts of recombinant glyceraldehyde-3-phosphate dehydrogenase protein (GAPDH; Sigma-Aldrich) were added to the reaction mix. Finally, pellets were resuspended in 10 μl of loading buffer and analyzed by Western blot.

### Cell Culture and Transfection

Cell lines expressing the different TDP-43 forms were previously described in Budini et al. (2015). Briefly, Human embryo kidney cell line 293 (HEK293) cell lines were grown in DMEM (Hyclone SH30243.02) supplemented with 10% fetal bovine serum (GIBCO) and antibiotic-antimycotic suspension (GIBCO 15240-062). When serum starvation (STV) was applied, cells were carefully washed with PBS for 2 or 3 times and then kept in DMEM 0% FBS plus Antibiotic Antimycotic. To generate the stable cell line overexpressing EGFP-Hsc70, HEK293 Flp-in cells were transfected with 0.5 μg of pcDNA5/FRT/TO GFP-HSPA8 plasmid (Addgene) plus 0.5 μg of pOG44 (Invitrogen). Stable integration was gradually selected using 100 μg/ml Hygromycin B (Invitrogen). All transgenes were induced with 1 μg/ml of tetracycline (Sigma-Aldrich). Transient transfections were carried out with 0.5 μg of plasmids using Effectene reagent (Qiagen).

### Lysosome Isolation From Cell Lines

The protocol for lysosomal isolation from cells was performed according to Storrie and Madden (1990) and Agarraberes et al. (1997) and was used by other authors to study CMA (Storrie and Madden, 1990; Terlecky and Dice, 1993). Specifically, for every cell line, 8 × 10^5^ cells were cultured in four 150 mm plates and after washing with cold PBS, they were scrapped with 2 ml of PBS and collected by centrifugation at 500 *g* for 5 min 4°C. Then cells were washed and finally resuspended in 2 ml of 2.5 M sucrose pH 7.2 and lysed by nitrogen cavitation. Samples were centrifuged at 2,500 *g* for 15 min 4°C and postnuclear supernatant (PNS) placed on the top of a Nycodenz gradient (2 ml 35% Nycodenz/0, 25 M Sucrose, 2 ml 17% Nycodenz/0, 25 M Sucrose, 3 ml Percoll/0, 25 M Sucrose) using a 14 × 89 mm ultra-clear tube. Samples were spin-down at 20,000 rpm for 35 min 4°C in an SW40.1 rotor. Lysosomal/Mitochondria interphase was collected and placed in a new ultra-clear tube 14 × 89 mm for mixing with 80% Nycodenz to achieve a density of 35%. A new gradient of Nycodenz was generated (2 ml 35% Lysosomal/Mitochondria sample, 2 ml 17% Nycodenz/0.25 M Sucrose, 2 ml 5% Nycodenz/0.25 M Sucrose) and new ultra-centrifugation was performed as described above. Mitochondria and lysosomal fractions were separated (first and second band from the bottom, respectively) and collected from the interphases generated. Samples were washed with one volume of 0.25 M sucrose and centrifuged at max velocity (4.500 *g*) for 15 min 4°C. The supernatant was discarded carefully, and pellets were used for Western blot analysis using the following antibodies: anti-TDP-43 (Proteintech 10782-2-AP), anti-Lamp2A (Abcam ab18528), anti-GAPDH (Santa Cruz sc-365062) and anti-Flag (Sigma M2).

### Cytosolic S100 Fraction Purification

S100 cytosolic fractions were prepared according to the protocol of Schneider et al. (2015). Cells at 80–90% of confluence were washed with cold PBS, scrapped with 4 ml of PBS and centrifuged at 350 *g* for 5 min 4°C. Pellets were vortexed in 1 ml of hypotonic buffer (20 mM HEPES pH 7.3, 5 mM CH_3_CO_2_K, 0.5 mM MgCl_2_) supplemented with protease inhibitor cocktail (Thermo Scientific), 0.5 mM DTT, 0.1 mM PMSF; and kept on ice for 10 min. Then, cells were disrupted by nitrogen cavitation and cell lysates centrifuged at 100,000 *g* for 70 min at 4°C (acceleration 4, deceleration 9) in the 70.1 Ti rotor. Supernatants were recovered for Western blot analysis.

### Co-immunoprecipitation Assay

Cells were collected in RIPA lysis buffer (50 mM Tris/HCl pH 7.4, 150 mM NaCl, 1% NP-40, 0, 1% SDS, 1 mM EDTA pH 8, 1 mM PMSF, 0, 5% Sodium Deoxycholate) supplemented with protease inhibitors (Thermo Scientific A32965) and incubated 30 min on ice. After centrifuging at 500 *g* at 4°C, supernatants were quantified. One milligram (1 mg) of total protein from cell lysate was incubated with 40 μl of Anti-Flag M2 Affinity Gel (Sigma-Aldrich) overnight at 4°C. Then, beads were centrifuged and washed with PBS three times for 10 min at 4°C and then resuspended in 50 μl of resuspension buffer (50 mM Tris/HCl pH 7.4, 5 mM EDTA, 10 mM DTT, 1% SDS) plus 20 μl of SDS 5×. Western blot analysis was performed by using anti-Flag (Sigma-Aldrich, M2), anti-Hsc70 (Thermo Scientific, 13D3) and anti-Lamp2A (Abcam ab18528).

### Western Blot Assay

Proteins were run in 10% SDS-Poly acrylamide gel and transferred to nitrocellulose membrane using a Trans-Blot Turbo System-Bio-Rad (only when LC3 II protein was analyzed, a 12% SDS-Poly acrylamide gel was used). To evaluate samples from lysosomal isolation and cell lysates, 10 μg to total protein was loaded on the gel. The nitrocellulose membrane was incubated with a blocking solution (TBS 0.1% Tween-20, 5% BSA) for 1 h. First antibodies were diluted in a TBS 0.1% Tween-20, 2% BSA solution and incubated overnight at 4°C. After three washes, the membrane was incubated with secondary antibodies in a TBS 0.1% Tween-20 solution for 1–2 h at room temperature. After three washes, the membrane was developed in an Odyssey-FC system (Licor). Antibodies and their working solutions were the follow: anti-TDP-43 (Proteintech #10782-2-AP, 1:2,000), anti-Lamp2A (Abcam #ab18528, 1:2,000) and anti-GAPDH (Santa Cruz #sc-365062, 1:5,000), anti-Hsc70 (Thermo Scientific #13D3, 1:5,000), anti-Flag (Sigma-Aldrich M2, 1:1,000), anti-LC3 A/B (Cell Signaling #4108, 1:1,000), p62 (Abnova #H00008878-M01, 1:5,000), BAG3 (Novus Biological # NBP2-27398ss, 1:2,000).

### RT-qPCR

Total RNA was extracted by using Trizol (Invitrogen) according to the manufacturer’s instructions, treated with DNase I (Invitrogen) and then quantified. Reverse transcription was performed using 1 μg of total RNA and random primers (Thermo Scientific) in 20 μl of the total volume of the reaction mixture using the reverse transcription reagents kit (Invitrogen). For PCR, KAPA SYBR FAST qPCR (KAPABIOSYSTEMS) kit was used. Reactions were made in triplicate using 2 μl of cDNA and 0.4 μM final concentration of each primer in 25 μl final volume. Primers were the following: β-actin, sense (5′-GATCTGGCACCACACCTTCT-3′) and antisense (5′-GGGGTGTTGAAGGTCTCAAA-3′); hHsc70, sense (5′-GGAGGTGGCACTTTTGATGT-3′) and antisense (5′-AGCAGTACGGAGGCGTCTTA-3′); hLamp2A, sense (5′-TATGTGCAACAAAGAGCAGA-3′) and antisense (5′-CAGCATGATGGTGCTTGAGA-3′). Reactions were subjected to dissociation curve analysis to exclude the possibility of nonspecific amplification. Changes for each gene were calculated using the mean of the change in Ct values (ΔCt) normalized to the Ct values of β-actin for each sample (2^−ΔΔCt^).

### Immunofluorescence Microscopy

Cells were fixed in cold methanol (−80°C) for 5 min on ice, washed with PBS and then incubated with a blocking solution (2% BSA in PBS 1X) for 30 min. Primary antibodies were incubated in blocking solution at the following concentrations: anti-Flag, 1:250 (Sigma M2); anti-Lamp2A 1:200 (Abcam); anti-Galectin 3 1:50 (Santa Cruz sc-23938). After washing with PBS 1X, cover-glasses were incubated with secondary antibodies 1 h with anti-mouse-Alexa Fluor 594 (A21203) or anti-rabbit-Alexa Fluor 488 (A21444). Finally, Hoechst 33342 was used for cell nuclei. Samples were imaged with a Nikon Eclipse Ti-E microscope and Fiji software.

### Statistics

Data analysis was performed from at least three independent experiments, obtained separately from different sets of cell cultures or animals. Different statistics methods were applied. When one group was analyzed, one-sample t and Wilcoxon test were used and numerical results reported as ±SEM. When more than two groups were compared, one or two-way ANOVA were used, followed by the Bonferroni *post hoc* test to determine statistical significance (*p* < 0.05). Numerical results are reported as mean ±SE. GraphPad software 8 was used to perform the statistical analysis. For Western blot densitometric analysis, the ImageJ software was used. ns: no significant, *p < 0.05, **p < 0.01, ***p < 0.001, ****p < 0.0001.

## Results

### TDP-43 as CMA Substrate *in vitro*

**Figure 1:**
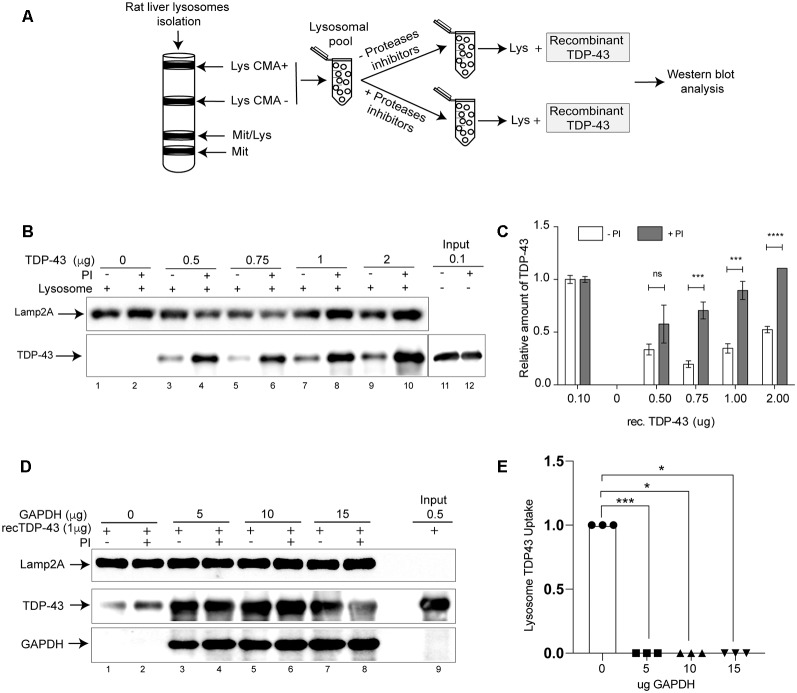
TAR DNA binding protein 43 kDa (TDP-43) is substrate of chaperone mediated autophagy (CMA) *in vitro*. **(A)** Schematic representation showing the isolation of lysosomal fractions from rat liver tissue and the subsequent analysis of samples by Western blot. **(B)** Lysosomal CMA+ and CMA− fractions were pooled, preincubated with or without proteases inhibitors (PI) and then recombinant TDP-43 was added at different concentrations (0, 0.5, 0.75, 1 and 2 μg). The presence of lysosomal associated membrane protein isoform 2 isoform A (Lamp2A) and TDP-43 was analyzed by Western blot. **(C)** Relative amounts of recombinant TDP-43 were quantified from at least three independent experiments and the input was used to normalize the samples. The proportion of TDP-43 in PI- and PI+ was calculated using normalized data. Numerical results are reported as mean ± SE. Differences among means were analyzed from three independent experiments using two-way ANOVA, followed by the Bonferroni *post hoc* test to determine statistical significance (*p* < 0.05). **(D)** Recombinant TDP-43 (1 μg) was preincubated with different amounts of recombinant glyceraldehyde-3-phosphate dehydrogenase protein (GAPDH; 0, 5, 10 and 15 μg) and subjected to lysosomal degradation. TDP-43, Lamp2A, and GAPDH were analyzed by Western blot. **(E)** Densitometric quantification of TDP43 protein levels showed in **(D)** were normalized by the Lamp2A signal. The up-take was considered as the difference between TDP-43 in PI+ and PI− (Up-take = PI+–PI−). Three independent experiments were quantified and analyzed by ordinary one-way ANOVA (*p* < 0.05). ns: no significant, *p < 0.05, **p < 0.01, ***p < 0.001, ****p < 0.0001.

Aberrant forms of different neurodegenerative associated proteins that have been recognized as CMA substrates are able to alter the activity of this pathway. To study whether this is the case for TDP-43, we first wanted to confirm if this protein is a real CMA substrate. Although TDP-43 has been proposed to be a CMA substrate (Huang et al., 2014), a battery of additional experiments need to be performed to confirm this fact (Kaushik and Cuervo, 2008, 2009, 2018; Patel and Cuervo, 2015). Thus, we used a well-established protocol (Cuervo and Dice, 1996; Cuervo et al., 1997) to evaluate if recombinant TDP-43 could be degraded by lysosomes isolated from rat liver. We isolated lysosomal fractions enriched in lysosomes with high (Lys CMA+) and low CMA activity (Lys CMA−; Figure 1A). Lysosomal fractions were pooled, pre-treated with or without proteases inhibitors (Lys +PI and Lys −PI, respectively) and then incubated with different amounts of recombinant TDP-43 (Figure 1A). Independently of the amount used, we could observe TDP-43 degradation by Lys −PI samples, but not by Lys +PI in intact lysosomes (Figures 1B,C), indicating that the protein is transported into lysosomes. In order to confirm that TDP-43 was specifically being degraded by CMA+ lysosomes by a receptor-mediated mechanism, we performed a competition assay using recombinant purified GAPDH (Glyceraldehyde 3-phosphate dehydrogenase), a well-known CMA substrate (Cuervo et al., 1994, 1995). This assay has been previously used to confirm the specific degradation of other proteins by CMA (Wang et al., 2009). Thus, we pre-incubated a constant amount of recombinant TDP-43 with increasing amounts of recombinant GAPDH protein (0, 5, 10 and 15 μg). The degradation of recombinant TDP-43 protein was observed in Lys −PI samples in the absence of GAPDH (Figure 1D, compare lines 1 and 2). On the contrary, a complete impairment of recombinant TDP-43 degradation was observed in all Lys −PI samples pre-incubated with GAPDH (Figures 1D,E). Altogether, these results confirm that TDP-43 is specifically degraded by CMA lysosomes *in vitro*.

### TDP-43 as CMA Substrate *in vivo*

**Figure 2:**
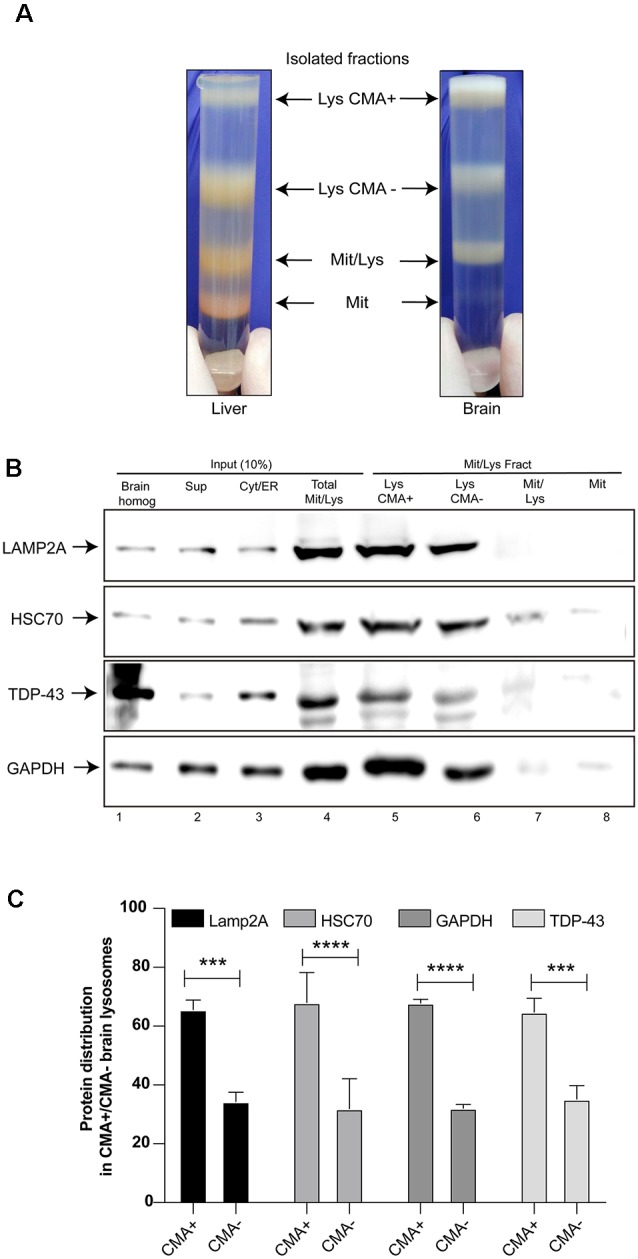
TDP-43 as a substrate of CMA *in vivo*. **(A)** Lysosomal fractions CMA+ and CMA− were isolated from rat brains in a similar way as indicated in Figure 1A. The different fractions obtained were compared with fractions isolated from rat liver. **(B)** CMA+ and CMA− lysosomal fractions were analyzed by Western blot in order to evaluate the protein levels of endogenous TDP-43, Lamp2A, Heat shock cognate protein 70 kDa (Hsc70) and GAPDH. **(C)** Densitometric quantification of each protein was performed in CMA+ and CMA− lysosomal fractions. Densitometric data obtained from CMA+ and CMA− conditions were summed and taken as 100%. Using this data, the proportions of each protein in CMA+ and CMA− lysosomal were calculated. The calculated proportions from three independent experiments were used to obtain the final graph. Numerical results are reported as mean ± SE. Differences among means were analyzed from three independent experiments using two-way ANOVA, followed by the Bonferroni *post hoc* test to determine statistical significance (*p* < 0.05). ***p < 0.001, ****p < 0.0001.

TDP-43 is directly associated with neurodegenerative diseases, thus it was important to evaluate if this protein can be a CMA substrate in brain tissues. For this, CMA+ and CMA− lysosomes were isolated from rat brain tissue and the samples were analyzed by Western blot. The brain fractions were similar to those obtained from the rat liver tissue, with the exception of the mitochondrial fraction band (Mit) that was thinner (Figure 2A). The analysis of Lamp2A, Hsc70 and GAPDH protein levels, showed a higher content of this CMA markers in the Lys CMA+ sample (around 60%), compared with Lys CMA− (around 40%), indicating an appreciable separation of the two lysosomal fractions (Figures 2B,C). Interestingly, endogenous TDP-43 was detected in both lysosomal fractions in similar proportions as observed for Lamp2A, Hsc70 and GAPDH (Figure 2B, lines 5 and 6 and Figure 2C). Thus, this result suggests the participation of TDP-43 as a CMA substrate *in vivo*, also in brain tissue.

### Inhibition of CMA Increases Protein Levels of Wild Type and an Aggregate-Prone Form of TDP-43

**Figure 3:**
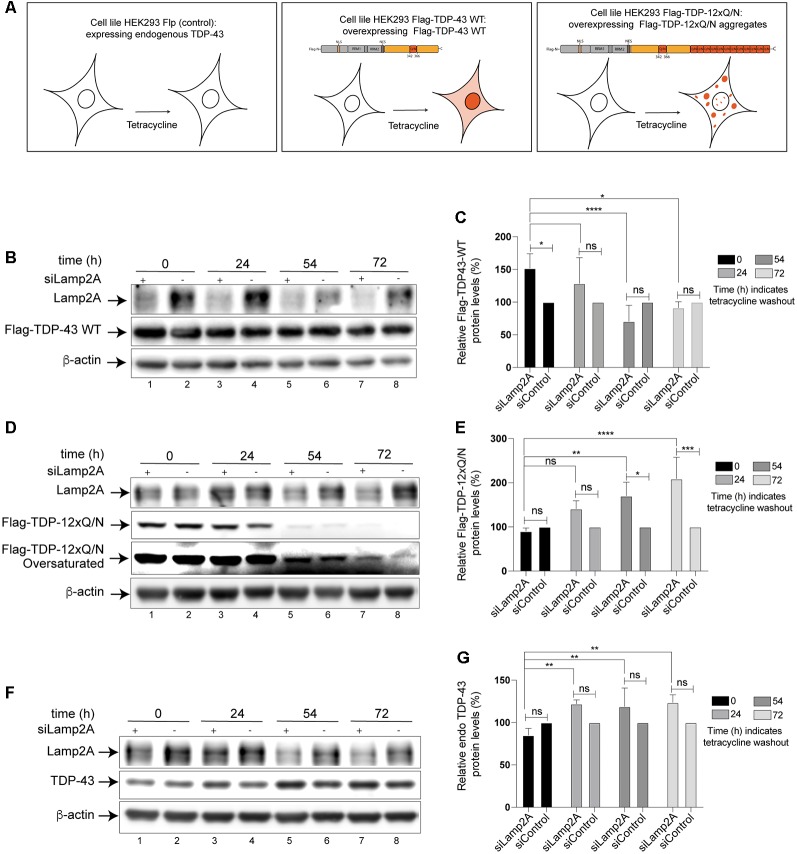
Protein levels of wild type and aggregated-prone forms of TDP-43 increase after inhibition of CMA. **(A)** Schematic representation of the three Human embryo kidney cell line 293 (HEK293) cell lines used in this study. HEK293 Flp-in (expressing endogenous TDP-43), HEK293 Flag-TDP-43 WT (overexpress a Flag- wild type form of TDP-43 after tetracycline induction), HEK293 Flag-TDP-12xQ/N (overexpress a Flag- aggregate-prone form of TDP-43 after tetracycline induction; Budini et al., 2015). **(B)** Lamp2A down-regulation was carried out in HEK293 Flag-TDP-43 WT cell line by transfecting an interfering RNA against human Lamp2A (siLamp2A). The expression of Flag-TDP-43 WT was induced for 48 h and then tetracycline was removed. The remaining levels of Flag-TDP-43 WT were evaluated by Western blot at indicated time points. An unrelated siRNA was used as control (see “Materials and Methods” section). Forty-eight hours post siRNA transfection, the expression of Flag-TDP-43 WT was evaluated at by Western blot at indicated time points. **(C)** Densitometric quantification of Flag-TDP-43 WT from **(B)**. **(D)** Lamp2A siRNA was transfected in HEK293 Flag-TDP-12xQ/N cell line as indicated in **(B)**. The expression of Flag-TDP-12xQ/N was induced for 48 h and then tetracycline was removed. The remaining levels of Flag-TDP-12xQ/N were evaluated by Western blot at indicated time points. **(E)** Densitometric quantification of Flag-TDP-12xQ/N from **(D)**. **(F)** Lamp2A siRNA was transfected in HEK293 Flp-in as indicated in **(B)** and the protein levels of endogenous TDP-43 were evaluated by Western blot at indicated time points. **(G)** Densitometric quantification of endogenous TDP-43 from **(F)**. Quantifications showed in **(C,E,G)** were calculated as follow: every time point was normalized against its own β-actin loading control. Upon normalization, every siLamp2A point was compared with the corresponding si-Control point (considered as 100%). Finally, every siLamp2A/siControl time point was compared with point zero (0) and between them. Numerical results are reported as mean ± SE. Differences among means were analyzed from three independent experiments using two-way ANOVA, followed by the Bonferroni *post hoc* test to determine statistical significance (*p* < 0.05). ns: no significant, *p < 0.05, **p < 0.01, ***p < 0.001, ****p < 0.0001.

The previous experiments confirmed that TDP-43 is a CMA substrate. However, up to date, nothing is known about the relationship between TDP-43 aggregation and CMA. To study this aspect, we used a cellular TDP-43 aggregation model previously reported. Briefly, this model is a tetracycline-inducible HEK293 cell line overexpressing a specific form of TDP-43 prone to aggregate (Flag-TDP-12xQ/N; Budini et al., 2015). In the experiments, HEK293 Flag-TDP-43 WT (expressing a Flag-TDP-43 WT form) and HEK293 Flp-in (expressing endogenous TDP-43) were used as control (Figure 3A).

First, we down-regulated CMA activity using a specific siRNA for Lamp2A (siLamp2A) and evaluated the turnover of the different forms of TDP-43 indicated in Figure 3A (Flag-TDP-43 WT, Flag-TDP-12xQ/N and endogenous TDP-43). For this, cells expressing Flag-TDP-43 WT, Flag-TDP-12xQ/N and endogenous TDP-43 were incubated with tetracycline and transfected with siLamp2A. After 48 h, the tetracycline was washed out (time point 0) and the protein levels of TDP-43 were evaluated at the indicated time points. An additional pulse of siLamp2A was added at time points 24 h and 54 h to maintain the protein depletion (siLamp2A efficiency was around 60%, Supplementary Figure S1). As it can be observed, at different time points upon tetracycline washout, an increase in the protein levels of Flag-TDP-43 WT (Figures 3B,C), Flag-TDP-12xQ/N (Figures 3D,E) and endogenous TDP-43 (Figures 3F,G) was observed in Lamp2A down-regulated cells. Thus, these results indicate that CMA loss of function, through Lamp2A down-regulation, affects the turnover of endogenous TDP-43 as well as of the overexpressed Flag-TDP-43 WT and the aggregate-prone form Flag-TDP-12xQ/N.

### Wild Type and the Aggregated-Prone Form of TDP-43 Are Present in the Lysosomal Fractions Isolated From Cell Culture

**Figure 4:**
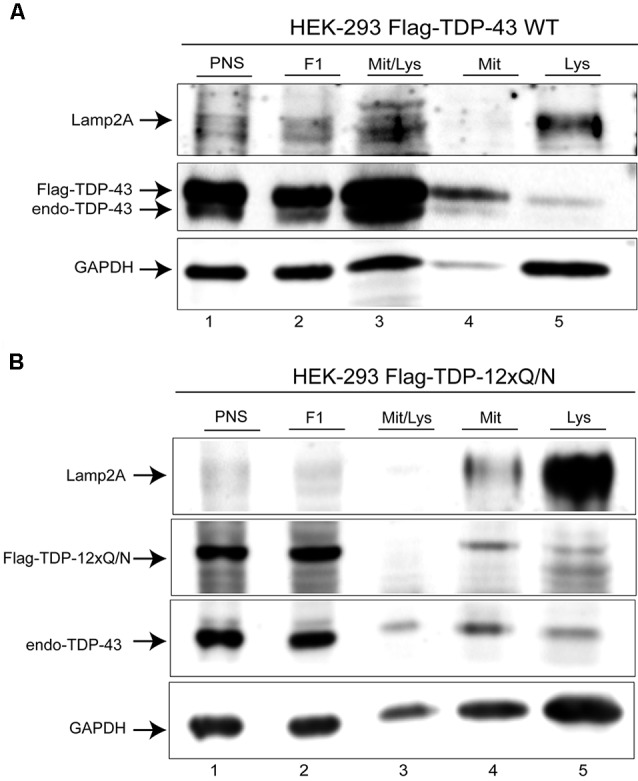
Wild type and aggregated-prone TDP-43 forms are present in lysosomal fractions isolated from cell culture. **(A)** The lysosomal fraction was isolated from cell line over-expressing Flag-TDP-43 WT during 72 h, then endogenous, as well as Flag-tagged protein was evaluated by Western blot. **(B)** The lysosomal fraction was isolated from cell line over-expressing Flag-TDP-12xQ/N during 72 h, then endogenous, as well as Flag-tagged protein was evaluated by Western blot. PSN, post-nuclear fraction; F1, post-nuclear fraction after 1st ultracentrifugation step; Mit/Lys, fraction containing mitochondria and lysosomes after 1st ultracentrifugation step; Mit, mitochondria fraction after 2nd centrifugation step; Lys, lysosomal fraction after 2nd centrifugation step. Western blots are representative from at least three independent experiments.

The previous experiments suggested that Flag-TDP-43 WT and the aggregated-prone form Flag-TDP-12xQ/N can be CMA substrates. To confirm this, we addressed whether both TDP-43 forms were present in CMA lysosomal fractions isolated from their respective cell lines. As shown, lysosomal fractions from both cell lines were enriched with Lamp2A and GAPDH proteins, indicating a high content of CMA positive lysosomes (Figures 4A,B, line 5). In these lysosomal samples, the Flag-TDP-43 WT protein (Figure 4A, line 5, upper band) and the aggregate-prone form Flag-TDP-12xQ/N were clearly detected (Figure 4B, line 5). Regarding the endogenous TDP-43 form, a faint band was observed in the cell line over-expressing Flag-TDP-43 WT (Figure 4A, line 5, lower band). Endogenous TDP-43 is downregulated in cells overexpressing Flag-TDP-43 WT (Avendaño-Vázquez et al., 2012), explaining why low levels of endogenous TDP-43 are observed in these samples. However, endogenous TDP-43 was clearly observed in the cell line over-expressing Flag-TDP-12xQ/N (Figure 4B, line 5). Altogether these results support the idea that TDP-43 WT and its aggregated-prone form are CMA substrates in cell culture.

### Aggregated-Prone Form of TDP-43 Interacts With Hsc70-CMA Component in Cell Culture

**Figure 5:**
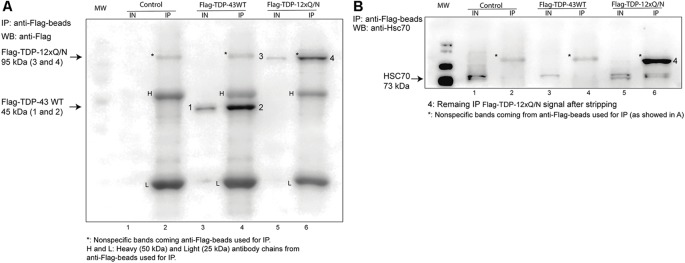
Aggregated-prone form of TDP-43 co-precipitates with Hsc70. **(A)** The HEK293 Flp-in, HEK293 Flag-TDP-43 WT and HEK293 Flag-TDP-12xQ/N cell lines were incubated with tetracycline for 72 h. Then, immunoprecipitation was performed using an Anti-Flag M2 Affinity Gel. Immunoprecipitated Flag-tagged proteins were analyzed by Western blot using an anti-Flag M2 antibody. **(B)** The presence of co-precipitated Hsc70 protein in samples shown in **(A)** was evaluated by Western blot using an anti-Hsc70 antibody. IN, inputs (20 μg of cell lysate); IP, immunoprecipitates. Western blots are representative from at least three independent experiments.

**Figure 6:**
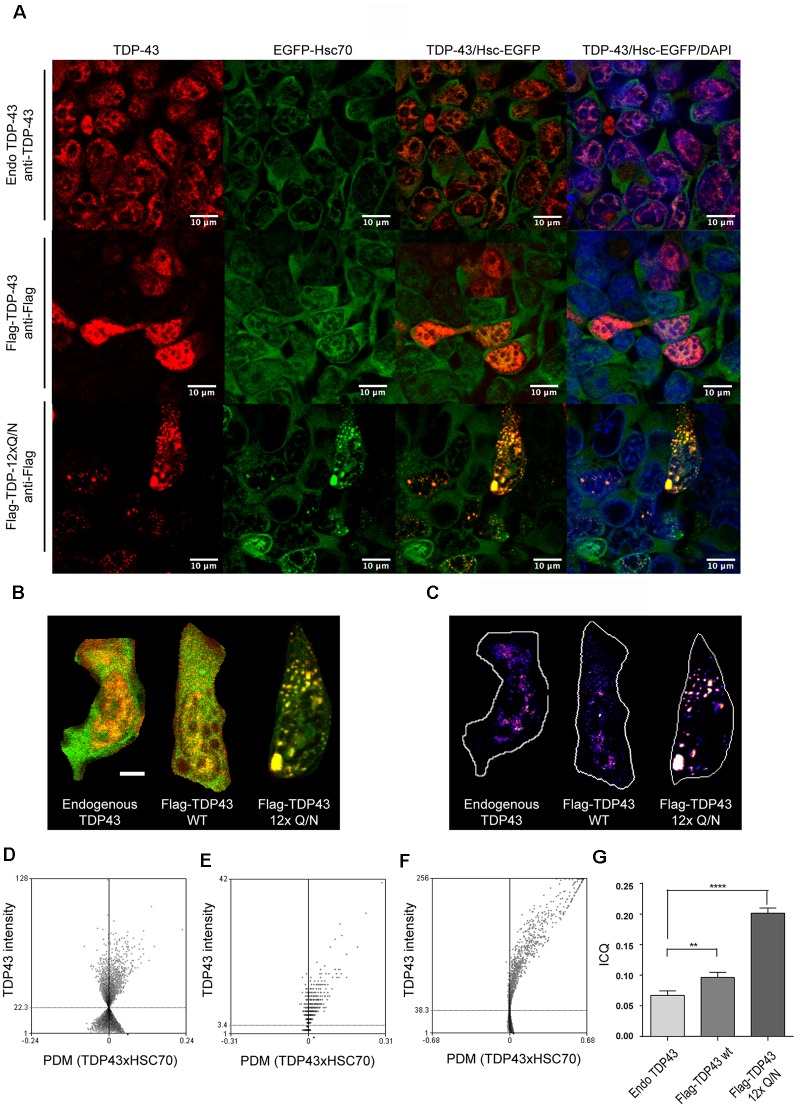
Aggregated-prone form of TDP-43 co-localizes with Hsc70.** (A)** HEK293 stably transfected with a plasmid expressing the EGFP-Hsc70 fusion protein were incubated with tetracycline for 24 h to allow the overexpression of the EGFP-Hsc70 protein. After tetracycline stimulation, the cell line was transiently transfected for 48 h with plasmids expressing Flag-TDP-43 WT, Flag-TDP-12xQ/N or the corresponding empty plasmid (pcDNA5 FRT/TO). Next, an immunofluorescence assay was performed to evaluate the co-localization of EGFP-Hsc70 with endogenous TDP-43 or the flagged proteins (Flag-TDP-43 WT and Flag-TDP-12xQ/N). Used antibodies were the following: anti-TDP-43 antibody (red, upper panels) an anti-Flag antibody (red, middle and lower panels). **(B)** Merged images used to calculate the spatial distribution of representative co-localizing pixels shown in **(C)** according to intensity correlation analysis (ICA) method. Positive PDM values (product of the differences from the mean) from the ICA and image pairs (scale bar = 5 μm) were calculated **(D–F)**. Representative ICA graphs obtained for the ICA of endogenous TDP43 and EGFP-HSC70 **(E)**, Flag-TDP43-WT and EGFP-HSC70 and TDP43 12xQ/N and HSC70-GFP.** (G)** Bars indicate mean intensity correlation quotients (ICQs) obtained from the analysis of pairs of immunofluorescence images for endogenous TDP-43 and EGFP-HSC70, Flag-TDP43-WT and EGFP-HSC70, and TDP43 12xQ/N and EGFP-HSC70. For all samples *n* ≥ 100. Ordinary one-way ANOVA was used (*p* < 0.05). **p < 0.01, ****p < 0.0001.

Then, we characterized whether wild type or TDP-43 aggregated-prone form were able to interact with the CMA the component Hsc70. For this, the control cell line, or the ones expressing Flag-TDP-43 WT or Flag-TDP-12xQ/N, were induced with tetracycline and then subjected or not to serum deprivation (+STV and −STV, respectively). We used serum deprivation condition as a CMA activity activator (Kaushik and Cuervo, 2009; Koga et al., 2011b; Patel and Cuervo, 2015) and with the aim of enhancing the interaction between the TDP-43 forms and the Hsc70 protein. Immunoprecipitation of both overexpressed TDPs forms (Flag-TDP-43 WT and Flag-TDP-12xQ/N) was efficiently carried out with an anti-Flag antibody (Figure 5A and Supplementary Figure S2A). Interestingly, compared with Flag-TDP-43 WT and the control cell line, co-precipitation of Hsc70 was only detected with the TDP-43 aggregated-prone form (Flag-TDP-12xQ/N) under −STV and +STV conditions (Figure 5B and Supplementary Figure S2B, respectively). Next, we wanted to confirm the interaction of the TDP-43 aggregated form with Hsc70. To test this, we generated a stable cell line that overexpressed EGFP-Hsc70 fusion protein under the tetracycline control. After tetracycline stimulation, the cell line was transiently transfected with plasmids expressing Flag-TDP-43 WT, Flag-TDP-12xQ/N or empty plasmid pcDNA5 FRT/TO. Next, the co-localization of EGFP-Hsc70 with endogenous TDP-43 or Flag-tagged proteins was analyzed by immunofluorescence. In addition, co-localization measurements were performed in immunofluorescence image pairs of segmented cells by intensity correlation analysis (ICA) method using the ICA plugin of ImageJ software. ICA allows the quantification of the co-localization of two bidimensional events in a heterogeneous background situation, in terms of the synchrony or dependency of their changes in intensity with respect to the mean of intensity of their respective signals (Bolte and Cordelières, 2006). ICQ values obtained with this analysis are distributed between −0.5 and +0.5; representing random staining when ICQ ~0; segregated staining when 0 > ICQ < −0.5; and dependent staining when 0 < ICQ < +0.5, and perfect co-localization when ICQ = 0.5. By applying these analyses, the co-localization between EGFP-Hsc70 and endogenous TDP-43 was practically undetected (Figures 6A–D). However, a discrete co-localization between EGFP-Hsc70 and Flag-TDP-43 WT could be observed (Figures 6A–C,E). Interestingly, and supporting the results obtained in the IP experiment, the aggregate-prone form of TDP-43 strongly co-localized with EGFP-Hsc70 (Figures 6A–C,F). When compared together, the aggregate-prone was the form that higher co-colocalized with EGFP-Hcs70 (Figure 6G). Overall these results indicate that, in the studied cell lines, preferentially the aggregation-prone form TDP-12xQ/N is able to interact with the CMA component Hsc70.

However, Hsc70 has also been implicated in the degradation of protein aggregates through macroautophagy by interacting with the co-chaperone Bcl-2-associated athanogene 3 protein (BAG3; Gamerdinger et al., 2009, 2011). Thus, additionally, we performed an immunoprecipitation under the same conditions showed in Figure 5 and evaluated the co-interaction of the aggregated-prone form of TDP-43 with BAG3, p62, and LC3B. Interestingly, Hsc70, BAG3, p62, and LC3B II co-precipitated only in the aggregation condition (Supplementary Figures S3A–E), suggesting that macroautophagy could be also implicated in the degradation of this aggregated-prone form of TDP-43. Despite this, and as mentioned below, the activation of macroautophagy was not evident along different time points of the aggregation induction (Supplementary Figures S5A–E). Thus, further experiments need to be done to understand which is the contribution of macroautophagy in regulating the turnover of this aggregated-prone form of TDP-43.

### TDP-43 Aggregation Up-Regulates CMA Activity

**Figure 7:**
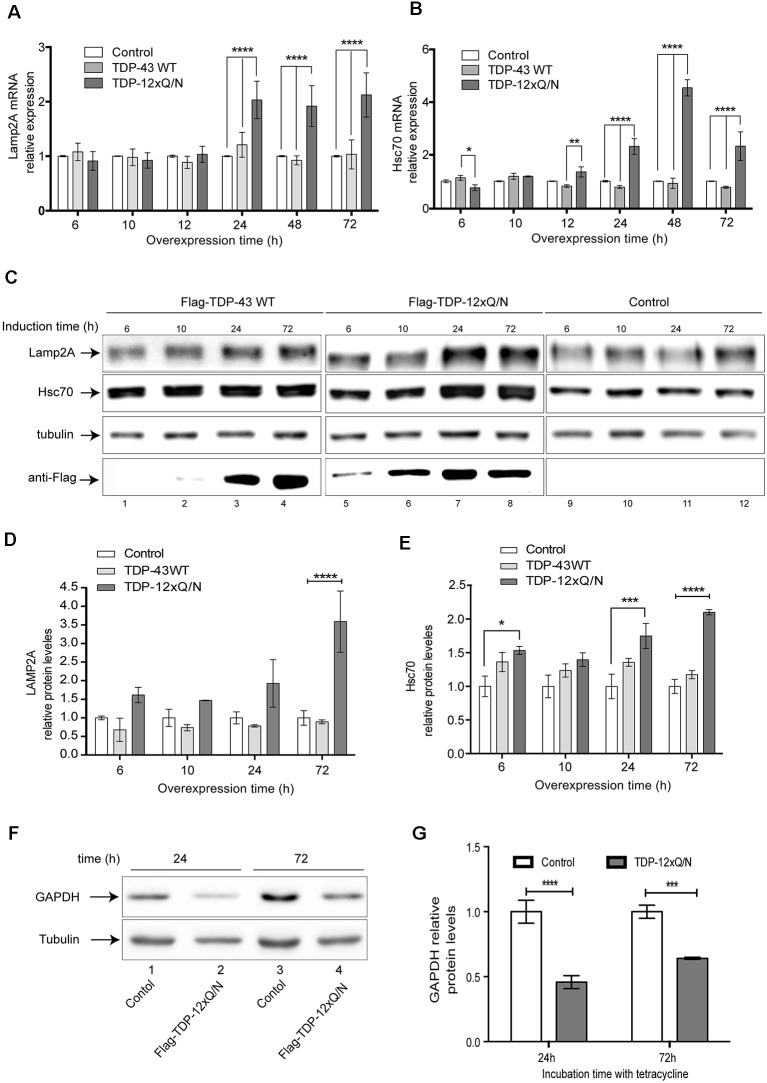
TDP-43 aggregation up-regulates Lamp2A and Hsc70 expression. The HEK293 Flp-in, HEK293 Flag-TDP-43 WT and HEK293 Flag-TDP-12xQ/N cell lines were incubated with tetracycline at indicated time points.** (A)** Relative Lamp2A and **(B)** Hsc70 mRNA levels were evaluated by RT-qPCR. Changes for each gene were calculated using the mean of the change in Ct values (ΔCt) normalized to the Ct values of β-actin for each sample (2^−ΔΔCt^). Graphics were performed using the mean of 2^−ΔΔCt^ from three independent experiments. Statistics were performed using ANOVA two-way. Numerical results are reported as mean ± SE. **(C)** The HEK293 Flp-in, HEK293 Flag-TDP-43 WT and HEK293 Flag-TDP-12xQ/N cell lines were incubated with tetracycline at indicated time points. Then, Lamp2A and Hsc70 protein levels were evaluated by Western blot. **(D)** Densitometric quantification of Lamp2A from **(C)**. **(E)** Densitometric quantification of Hsc70 from **(C)**. **(F)** Soluble S100 fractions were isolated from HEK293 Flp-in control cell line or cells over-expressing Flag-TDP-12xQ/N during 24 h and 72 h. Then, protein levels of GAPDH were evaluated by Western blot. **(G)** Densitometric quantification of GAPDH protein levels showed in **(F)**. Quantifications showed in **(D,E,G)** were calculated as follow: every time point was normalized against its own tubulin loading control. Upon normalization, each point was compared with the corresponding control point (considered as 1). Numerical results are reported as mean ± SE. Differences among means were analyzed from three independent experiments using two-way ANOVA, followed by the Bonferroni *post hoc* test to determine statistical significance (*p* < 0.05). *p < 0.05, **p < 0.01, ***p < 0.001, ****p < 0.0001.

To our knowledge, there is no report about the effects produced by TDP-43 aggregation on CMA activity. To study this, we evaluated if TDP-43 aggregation can modify Lamp2A and Hsc70 expression at different time points (0, 6, 12, 24 48 and 72 h) after aggregates induction. Increased Lamp2A and Hsc70 mRNA levels were observed in the cell line harboring TDP-43 aggregates after 24 h (Figures 7A,B). Interestingly, no changes in Lamp2A or Hsc70 mRNA levels were observed in Flag-TDP-43 WT or control cell lines. Moreover, similar to what we observed for the mRNA levels, Lamp2A and Hsc70 protein levels also increased only in TDP-43 aggregates producing cells (Figures 7C–E). Overall, these results indicate that in this model an up-regulation of the principal CMA components, Lamp2A and Hsc70, specifically occurs in response to the overexpression of the aggregation-prone form of TDP-43.

To confirm whether the presence of TDP-43 aggregates can enhance the CMA activity, we investigated GAPDH protein levels, a well-known CMA substrate, in cells expressing TDP-43 aggregates during 24 and 72 h. Compared with the control cell line, a reduction in GAPDH protein levels was observed in the cells expressing aggregates (Figures 7F,G). Furthermore, this decrease was more evident at 24 h than at 72 h (Figure 7F, compare lines 2 with 4). In addition, changes in GAPDH mRNA levels were non-significant at 24 h post-aggregation. However, GAPDH mRNA levels augmented around 5 times at 72 h of aggregation induction, probably to supply the lack of GAPDH protein levels as a consequence of CMA activation (Supplementary Figure S4). We also studied whether macroautophagy activity could be altered in this aggregation model. For this, we induced Flag-TDP-43 or the aggregated-prone form Flag-TDP-12xQ/N at different time points (0, 6, 12, 24 48 and 72 h) and evaluated the macroautophagy markers p62 and LC3B by Western blot (Supplementary Figures S5A–E). Under the studied conditions, and compared with the control cell line, macroautophagy activity was not altered by the aggregation induction (Supplementary Figures S5C–E). However, although non statistically significant, we only observed an apparent macroautophagy activation with the overexpression of Flag-TDP-43 WT (Supplementary Figures S5B,D,E). Altogether, these results indicate that TDP-43 aggregation up-regulates CMA activity.

### TDP-43 Aggregation Affects Lamp2A-Positive Lysosomes

**Figure 8:**
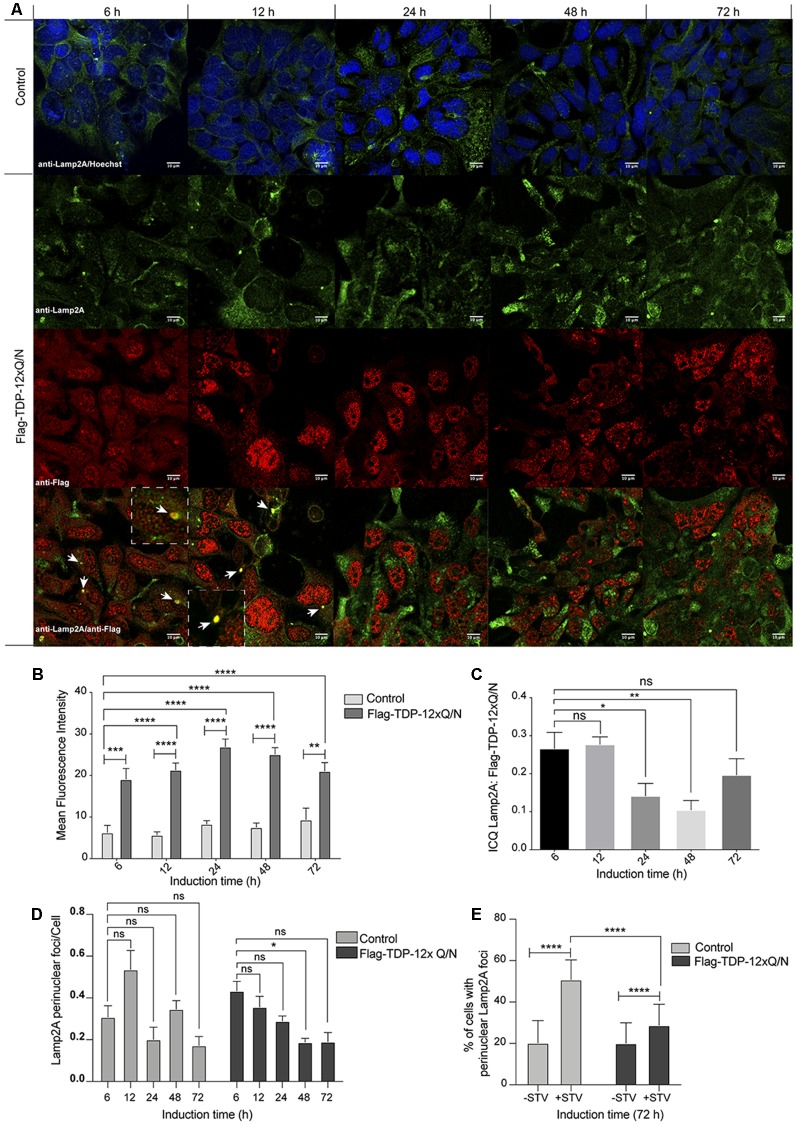
Subcellular distribution of Lamp2A along different time points of TDP-43 aggregation. **(A)** The HEK293 Flp-in cell or HEK293 Flag-TDP-12xQ/N cell lines were incubated with tetracycline at indicated time points. Next, an immunofluorescence assay was performed to evaluate the distribution pattern of Flag-TDP-12xQ/N protein and Lamp2A. Used antibodies were the following: anti-Flag antibody (red) and anti-Lamp2A antibody (green). At 6 and 12 h white arrow indicate the co-localization (yellow) between Flag-TDP-12xQ/N and Lamp2A. Dotted white lines insets show a magnification of one co-localization event. Images are representative of three independent experiments. **(B)** Mean intensities of Lamp2A immunostaining showed in **(A)** were obtained using the ImageJ software. Graph representing mean ± SE compared by two way ANOVA plus Bonferroni post-test (*N* ≥ 6). **(C)** Mean intensity correlation quotients (ICQs) obtained from the ICA of pairs of immunofluorescence images for endogenous Lamp2A and Flag-TDP-12xQ/N after different times of expression induction. One-way ANOVA plus Bonferroni post-test was applied (*N* ≥ 6). **(D)** Relative number of cells with perinuclear Lamp2a localization vs. the total of cells. One-way ANOVA plus Bonferroni post-test was applied (*N* ≥ 6). **(E)** Control cell line, or cell line overexpressing Flag-TDP-12xQ/N, were incubated with tetracycline for 72 h and then subjected or not to serum deprivation for 20 h (STV). Perinuclear localization of Lamp2A was evaluated by immunofluorescence (green) and the number of cells with perinuclear accumulation of Lamp2A positive lysosomes was quantified. Statistics were performed using the ANOVA two-way test from at least three independent experiments. ns: no significant, *p < 0.05, **p < 0.01, ***p < 0.001, ****p < 0.0001.

Under a CMA activating stimulus, lysosomes are recruited to the perinuclear zone of the cell (Kaushik and Cuervo, 2009; Patel and Cuervo, 2015; Johnson et al., 2016). Thus, we evaluated the subcellular localization of Lamp2A at different time points (6, 12, 24 48 and 72 h) after aggregation induction (Figure 8A). First, supporting the up-regulation of Lamp2A mRNA and protein levels upon aggregation induction (Figures 7A–E), and compared with the control cell line, we observed an increase in the signal of Lamp2A from 6 h to 72 h of aggregation induction (Figures 8A,B). Surprisingly, Lamp2A protein co-localized with Flag-TDP-12xQ/N in perinuclear foci at the early stages of the aggregation induction (Figure 8A: 6 and 12 h, white arrows and Figure 8C). However, such co-localization was transient as it decreased from 24 h post aggregation induction (Figures 8A,C). Next, as performed in Figure 6, the co-localization between Flag-TDP-12xQ/N and Lamp2A was confirmed by ICQ analysis (Figure 8C). In addiction, compared with the control cell line, images analysis also indicated that the perinuclear localization of Lamp2A diminished along the aggregation induction. However, this result was not statistically significant (Figures 8A,D). In order to confirm if the perinuclear localization of Lamp2A is affected by the aggregation induction, Flag-TDP-12xQ/N cell line was induced for 72 h and then subjected or not to starvation (STV) to stimulates CMA. Under these conditions, the control cell line responded well to the STV stimulus however, under the aggregation condition there were less cells with accumulation of perinuclear Lamp2A foci (Figures 8E and Supplementary Figure S6). The latter results support that, in this model, the aggregation of TDP-43 induces Lamp2A overexpression and that CMA could be implicated in the degradation of the aggregated-prone form at early stages of the aggregation process. In addition, the results also suggest that prolonged exposure to TDP-43 aggregates, the perinuclear localization of Lamp2A can be affected. However, additional experiments need to be performed to further confirm this idea.

**Figure 9:**
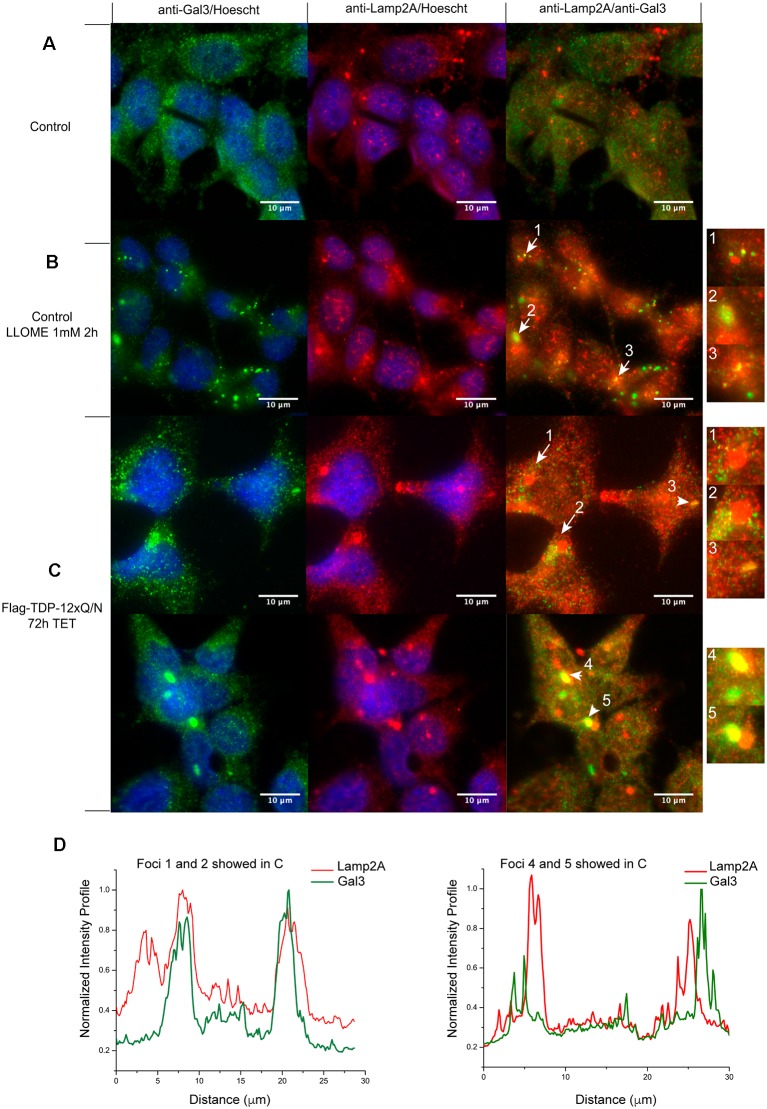
Lysosomal damage in TDP-43 aggregates producing cells. Subcellular localization of Galectin 3 (Gal3) was evaluated by immunofluorescence. **(A)** Control HEK293 cell line without 1 mM of LLOMe for 2 h. **(B)** Control HEK293 cell line with 1 mM of LLOMe for 2 h. Green Gal3 puncta observed (left panel) were considered as lysosomal damage. Lamp2A positive lysosomes co-localization with Gal3 puncta were evaluated (Lamp2A; red and Gal3; green). Numbered arrows indicate Lamp2A positive lysosomes colocalizing with Gal3 puncta (**B**, right panel, yellow). **(C)** Subcellular localization of Gal3 was evaluated in the HEK293 cell line over-expressing Flag-TDP-12xQ/N for 72 h. Like in **(B)**, the observed green Gal3 puncta were considered as lysosomal damage (left panels). Numbered arrows indicate Gal3 puncta surrounding Lamp2A positive lysosomes (**C**, right panel, yellow), whereas numbered arrows heads indicate Gal3 puncta colocalizing with Lamp2A lysosomes (**C**, right panels, yellow). Images are representative of at least three independent experiments. **(D)** Intensity line profiles of foci 1, 2, 4 and 5 showed in **(C)** were obtained using the plot profile plugin of ImageJ software. Data were normalized against maximum intensity values in each channel.

Finally, in order to have more insights into the effect of TDP-43 aggregates on CMA associated lysosomes, we evaluated the lysosomal damage associated to Lamp2A (Wang et al., 2009; Freeman et al., 2013; Jiang et al., 2017). First, we used a control cell line to set up Galectin 3 (Gal3) as a lysosomal damage biomarker (Aits et al., 2015; Jiang et al., 2017). After chemical lysosomal damage with LLOMe, Gal 3 localization changed to a puncta distribution in treated cells (Figure 9, compare A with B). In some cases, Gal3 puncta co-localized with Lamp2A positive lysosomes (Figure 9B, right panel, yellow dots). Interestingly, after 72 h of aggregate induction, Gal 3 also showed a cytoplasmic puncta distribution in the Flag-TDP-12xQ/N expressing cell line (Figure 9C, green dots). In addition, we observed Gal3 puncta surrounding Lamp2A positive lysosomes (Figure 9C, yellow dots indicated by arrows and Figure 9D) or directly co-localizing with them (Figure 9C, yellow dots indicated by arrows heads and Figure 9D). This result supports the idea that TDP-43 aggregation can also affect the integrity of lysosomes associated with CMA.

## Discussion

One of the main features of ALS and FTLD, two fatal and aging-dependent neurodegenerative diseases, is the abnormal aggregation of TDP-43 protein in affected neurons. On the other hand, the activity of CMA, a lysosomal-dependent degradation protein pathway, has been shown to decline with aging (Schneider and Cuervo, 2013; Schneider et al., 2015). Additionally, a subset of proteins associated with neurodegenerative processes are CMA substrates, and their aggregation can alter the CMA performance (Vogiatzi et al., 2008; Wang et al., 2009, 2016; Xilouri et al., 2009; Mak et al., 2010; Wang et al., 2010; Koga et al., 2011a; Qi et al., 2012; Orenstein et al., 2013; Caballero et al., 2018).

It has been shown that full-length TDP-43 protein levels and its 25 kDa and 35 kDa fragments can be differentially regulated by macroautophagy and the proteasome system (Wang et al., 2010; Scotter et al., 2014). Regarding CMA, Huang et al. (2014) characterized a KFERQ-like domain in TDP-43 and gave the first indications towards a possible TDP-43 regulation by CMA. More recently, another group, proposed a mechanism by which using a TDP-43 specific antibody could force the degradation of the misfolded protein through the CMA pathway (Tamaki et al., 2018). However, the connection between CMA and TDP-43 both, at the physiological and pathological levels, still poorly understood.

Here, we show that recombinant TDP-43 is degraded by lysosomes isolated from rat liver. TDP-43 degradation was competed by a *bona fide* CMA substrate like GAPDH, supporting the specificity of TDP-43 as a CMA substrate. Endogenous TDP-43 was also detected in the CMA+ lysosomal fraction isolated from rat brain thus, suggesting that TDP-43 can be also a CMA substrate *in vivo*. To our knowledge, this is the first report detecting endogenous TDP-43 in CMA+ lysosomal fractions from the brain and it supports the idea that TDP-43 protein levels could be regulated by CMA in this tissue. In addition to lysosomes isolated from mouse tissues, our experiments from cell culture, where Lamp2A was down-regulated and lysosomes were isolated, supported the fact that wild type forms of TDP-43 are CMA substrates.

We also show that CMA could control the protein levels of an aggregate-prone form of TDP-43. However, CMA protein substrates need to be unfolded in order to be up-taken by the lysosome thus, protein aggregates would not be good substrates of CMA (Dice, 2007; Bandyopadhyay and Cuervo, 2008). Thus, the increased protein levels of the aggregation-prone form TDP-12xQ/N observed after Lamp2A down-regulation, or its presence in lysosomes isolated from cell culture, can be related with a monomeric or oligomeric precursor rather than an aggregated form. This idea is supported by our results showing that Lamp2A only co-localized with Flag-TDP-12xQ/N at early periods of the aggregation process. During these periods of time, the Flag-TDP-12xQ/N protein had high and diffuse cytoplasm distribution (Figure 8A), suggesting that monomers or oligomers of Flag-TDP-12xQ/N are more prominent to be degraded by CMA. A recent work published by Wu et al. (2019) showed a similar result with α-synuclein. In this work, the authors demonstrated that disaggregation of α-synuclein by chemical compounds increases its localization with Lamp2A and make it more degradable by CMA. Thus, our results suggest that CMA would be participating in the control of the proteostatic equilibrium of TDP-43 at the initial steps of its aggregation.

Aberrant forms of other neurodegenerative proteins can affect the CMA activity (Vogiatzi et al., 2008; Koga et al., 2011a) however, no evidence has been reported about the connection between TDP-43 aggregation and CMA. Interestingly, in our model, the induction of TDP-43 aggregation triggered the up-regulation of Lamp2A and Hsc70 both, at the mRNA and protein levels. The latter was accompanied by a reduction in GAPDH protein levels. Although additional experiments need to be performed to determine the exact mechanism by which this is occurring, these results indicate that CMA is activated in response to a TDP-43 aggregation condition. Other proteins prone to aggregate also up-regulate CMA activity (Mak et al., 2010; Koga et al., 2011a) thus, it would be a common mechanism for many aggregate-prone proteins which are CMA substrates.

Aberrant forms of aggregate-prone proteins such as Tau and α-Syn have been shown to cause lysosomal damage associated with a CMA dysregulation (Wang et al., 2009; Xilouri et al., 2009; Freeman et al., 2013). In line with this, our results also showed that long periods of TDP-43 aggregation could cause a decrease in the perinuclear localization of Lamp2A-positive lysosomes, probably affecting their function. Supporting this, we found partial damage of lysosomes associated to Lamp2A in cells expressing TDP-43 aggregates. This suggests that CMA could be negatively affected after prolonged periods of TDP-43 aggregate exposure. However, additional experiments need to be performed to confirm this idea.

## Conclusion

This work clearly demonstrates that TDP-43 is a CMA substrate *in vitro* and probably *in vivo*. CMA can control the protein levels of wild type but also of an aggregate-prone form of TDP-43. Our results also indicate that a TDP-43 aggregation condition up-regulates CMA components (Lamp2A and Hsc70) and CMA activity, but also causes a partial damage of Lamp2A positive lysosomes, suggesting a general dysfunction of the pathway.

Although nothing is known regarding the state of CMA in ALS or FTLD patients, where TDP-43 aggregation is implicated, some reports have shown that Lamp2A and Hsc70 protein levels are altered in other neurodegenerative diseases (Papagiannakis et al., 2015; Klaver et al., 2018; Loeffler et al., 2018). Thus, our work supports the idea that a dysregulation in CMA would directly have an impact on TDP-43 homeostasis, contributing to the pathological mechanism of TDP-43 aggregation. On the other way around, our work suggests that an alteration in CMA activity could occur as a consequence of TDP-43 aggregation, triggering a generalized cellular stress that could contributes to the neuropathological process.

## Data Availability Statement

The datasets generated for this study are available on request to the corresponding author.

## Ethics Statement

The animal study was reviewed and approved by Bioethics Committee of Fundación Ciencia and Vida.

## Author Contributions

FO performed most of the experiments and helped with writing and discussion. JH helped with lysosomal purification and lysosomal transport assays experiment, and helped with discussion. FR performed qPCR experiments. JM performed and analyzed the perinuclear localization of lysosomes. JR performed the lysosomal damage experiment. AC contributed to the discussion. AA contributed with writing, editing and discussion. IA contributed with reagents, experimental design, image analysis, writing and discussion. MB wrote the article, made the figures, guided the experimental approaches and project. All authors read and approved the final manuscript.

## Conflict of Interest

The authors declare that the research was conducted in the absence of any commercial or financial relationships that could be construed as a potential conflict of interest.

## Abbreviations

ALS: Amyotrophic Lateral Sclerosis
FTLD: Frontotemporal Lobar Degeneration
TDP-43: TAR DNA binding protein 43 kDa
Ma: Macroautophagy
eMi: Endosomal Microautophagy
CMA: Chaperone Mediated Autophagy
MVBs: Multivesicular Bodies
Hsc70: Heat shock cognate protein 70 kDa
Lamp2A: lysosomal associated membrane protein isoform 2 isoform A
PD: Parkinson Disease
AD: Alzheimer Disease
HD: Huntington Disease
α-Syn: alpha-synuclein protein
Tau: microtubule-associated Tau protein
LRRK2: leucine-rich repeat kinase 2
PARK7: Parkinson disease protein 7 or protein deglycase DJ-1
Htt: Huntingtin protein
GAPDH: glyceraldehyde-3-phosphate dehydrogenase protein
EGFP: Enhanced Green Fluorescent Protein
Gal3: Galectin 3 protein
HEK293: Human embryo kidney cell line 293
SVT: serum deprivation or starvation
BAG3: co-chaperone Bcl-2-associated athanogene 3 protein
LC3B: Microtubule Associated Protein 1 Light Chain 3 Beta
p62: SQSTM1 (Sequestosome 1) or Phosphotyrosine-Independent Ligand For The Lck SH2 Domain of 62 KDa.
