# Supplementary material for: Chaperone Mediated Autophagy Degrades TDP-43 Protein and Is Affected by TDP-43 Aggregation
Source: Front Mol Neurosci. 2020 Feb 18;13:19. doi: 10.3389/fnmol.2020.00019 (PMC7040037; doi:10.3389/fnmol.2020.00019)
Supplement: Supplementary file 1 [file Data_Sheet_1.docx]

**Supplementary figure 1**

**Supplementary figure 2**

**Supplementary figure 3**

**Supplementary figure 4**


**Supplementary figure 5**

**Supplementary figure 6**

**Supplementary figure 1: siRNA Lamp2A efficiency**

Efficiency of siRNA against Lamp2A showed in figure 3 was calculated for every time point. Three independent experiments were used. Lamp2A protein levels were calculated by densitometry in siLamp2A or siControl conditions and every point was normalized by their corresponding β-actin loading control. Normalized data from siControl was considered as 100 %. Numerical results are reported as mean ±SEM. Differences among means were analyzed from three independent experiments using One sample t and Wilcoxon test, to determine statistical significance (p < 0.05). ns: no significant, *: p < 0.05, **: p < 0.001, ****: p < 0.0001

**Supplementary figure 2: Aggregated-prone form of TDP-43 co-precipitates with Hsc70 under starvation condition**

(A) The HEK293 Flp-in, HEK293 Flag-TDP-43 WT and HEK293 Flag-TDP-12xQ/N cell lines were incubated with tetracycline for 52 h and serum deprivation (starvation, +STV) was conducted for another 20 h in presence of tetracycline. Then, an immunoprecipitation was performed using an Anti-Flag M2 Affinity Gel. Immunoprecipitated Flag-tagged proteins were analyzed by Western blot using an anti-Flag M2 antibody. (B) The presence of co-precipitated Hsc70 protein in samples shown in A was evaluated by Western blot using an anti-Hsc70 antibody. IN: inputs (20 μg of cell lysate). IP: immunoprecipitates. Western blots are representative from at least three independent experiments.

**Supplementary figure 3: Aggregated-prone form of TDP-43 co-precipitates with Hsc70, Bag3, p62 and LC3 II**

(A) The HEK293 Flp-in, HEK293 Flag-TDP-43 WT and HEK293 Flag-TDP-12xQ/N cell lines were incubated with tetracycline for 52 h and serum deprivation (starvation, +STV) was conducted for another 20 h in presence of tetracycline. Then, an immunoprecipitation was performed using an Anti-Flag M2 Affinity Gel. Immunoprecipitated Flag-tagged proteins were analyzed by Western blot using an anti-Flag M2 antibody. The presence of co-precipitated Hsc70 (B), Bag3 (C), p62 (D) and LC3 (E) proteins in samples shown in A was evaluated by Western blot. IN: inputs (20 μg of cell lysate). IP: immunoprecipitates. Western blots are representative from at least three independent experiments.

**Supplementary figure 4: GAPDH mRNA levels at 24 and 72 hours post TDP-43 aggregation induction**

The HEK293 Flp-in and HEK293 Flag-TDP-12xQ/N cell lines were incubated with tetracycline at indicated time points. Next, a RT-qPCR was performed to evaluate the GAPHD mRNA levels. Changes in the gene were calculated using the mean of the change in Ct values (ΔCt) normalized to the Ct values of 18S rRNA for each sample (2-ΔΔCt). Graphics was performed using the mean of 2-ΔΔCt from three independent experiments. Statistics were performed using ANOVA Two-way. Numerical results are reported as mean ±SE. ns: no significant, **: p < 0.01.

**Supplementary figure 5: Protein levels of p62 and LC3 II** **macroautophagy markers along different time points of TDP-43 aggregation induction**

(A) The HEK293 Flp-in, (B) Flag-TDP-43 WT and (C) HEK293 Flag-TDP-12xQ/N cell lines were incubated with tetracycline at indicated time points. Next p62, LC3 and flagged proteins were analyzed by Western blot. p62 and LC3 II protein levels at all time points was calculated by densitometry and was normalized by their corresponding β-actin loading control. Normalized data from three independent experiments was used to compare the levels of (D) p62 and (E) LC3 II in the different time points. Numerical results are reported as mean ±SE. Differences among means were analyzed using two-way ANOVA, followed by the Bonferroni post hoc test to determine statistical significance (p < 0.05). ns: no significant.

**Supplementary figure 6: Lamp2A perinuclear localization after 72 h of TDP-43 aggregation**

Representative images of quantification showed in figure 8 D. Control cell line, or cell line overexpressing Flag-TDP-12xQ/N, were incubated with tetracycline for 72 h and then subjected or not to serum deprivation for 20 h (STV). Perinuclear localization of Lamp2A was evaluated by immunofluorescence using an anti-Lamp2A antibody (green).
